# Avian Pathogenic *Escherichia coli* (APEC): An Overview of Virulence and Pathogenesis Factors, Zoonotic Potential, and Control Strategies

**DOI:** 10.3390/pathogens10040467

**Published:** 2021-04-12

**Authors:** Dipak Kathayat, Dhanashree Lokesh, Sochina Ranjit, Gireesh Rajashekara

**Affiliations:** Center for Food Animal Health, Department of Animal Sciences, The Ohio State University, Wooster, OH 44691, USA; kathayat.1@osu.edu (D.K.); dhanashree.1@osu.edu (D.L.); ranjit.5@osu.edu (S.R.)

**Keywords:** APEC, virulence, pathogenesis, zoonosis, antibiotic resistance, vaccines, virulence inhibitors, infections

## Abstract

Avian pathogenic *Escherichia coli* (APEC) causes colibacillosis in avian species, and recent reports have suggested APEC as a potential foodborne zoonotic pathogen. Herein, we discuss the virulence and pathogenesis factors of APEC, review the zoonotic potential, provide the current status of antibiotic resistance and progress in vaccine development, and summarize the alternative control measures being investigated. In addition to the known virulence factors, several other factors including quorum sensing system, secretion systems, two-component systems, transcriptional regulators, and genes associated with metabolism also contribute to APEC pathogenesis. The clear understanding of these factors will help in developing new effective treatments. The APEC isolates (particularly belonging to ST95 and ST131 or O1, O2, and O18) have genetic similarities and commonalities in virulence genes with human uropathogenic *E*. *coli* (UPEC) and neonatal meningitis *E*. *coli* (NMEC) and abilities to cause urinary tract infections and meningitis in humans. Therefore, the zoonotic potential of APEC cannot be undervalued. APEC resistance to almost all classes of antibiotics, including carbapenems, has been already reported. There is a need for an effective APEC vaccine that can provide protection against diverse APEC serotypes. Alternative therapies, especially the virulence inhibitors, can provide a novel solution with less likelihood of developing resistance.

## 1. Introduction

Avian pathogenic *Escherichia coli* (APEC), an extra-intestinal pathogenic *E. coli* (ExPEC), causes diverse local and systemic infections in poultry, including chickens, turkeys, ducks, and many other avian species [1]. The most common infections caused by APEC in chickens are perihepatitis, airsacculitis, pericarditis, egg peritonitis, salphingitis, coligranuloma, omphalitis, cellulitis, and osteomyelitis/arthritis; these are commonly referred as avian colibacillosis [2]. APEC also causes swollen head syndrome in chickens and osteomyelitis complex in turkeys [2]. Colibacillosis is one of the leading causes of mortality (up to 20%) and morbidity in poultry and also results in decreased meat (2% decline in live weight, 2.7% deterioration in feed conversion ratio) and egg production (up to 20%), decreased hatching rates, and increased condemnation of carcasses (up to 43%) at slaughter [1,3,4]. Furthermore, APEC is responsible for high mortality (up to 53.5%) in young chickens [4]. Taken together, along with the treatment expenses, APEC costs the poultry industry hundreds of millions of dollars in economic losses worldwide [5]. In the United States (US), it has been estimated that economic losses to the broiler industry can be as high as $40 million annually only due to carcass condemnation [6].

**Scheme 1:**
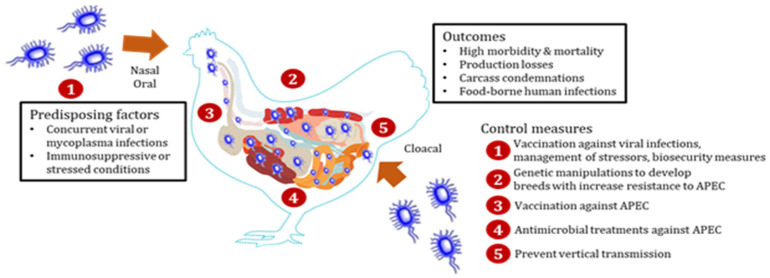
Schematic diagram showing overview of Avian pathogenic *Escherichia coli* (APEC) infection in chickens along with infection control checkpoints. After entry through oral, nasal, or cloacal routes, APEC colonizes the mucosal sites of gastrointestinal, respiratory, and reproductive tracts without causing disease in chickens. In the presence of concurrent viral or mycoplasma infections or under immunosuppressive or stressed conditions, APEC invades the mucosal layers and reach extra-intestinal organs (heart, liver, lung, spleen, kidney, reproductive organs, etc.) resulting in multi-systemic infections, which are commonly referred to as colibacillosis. Colibacillosis leads to high morbidity and mortality, production losses, and condemnation of carcasses as well as foodborne transmission risk to humans. Colibacillosis can be prevented by the management of stressors, biosecurity measures, and vaccination against APEC or associated viral infections. Chicken breeds with high intrinsic resistance to APEC can be developed through genetic technologies. Vertical transmission of APEC from breeders through contaminated eggs should be monitored to prevent APEC entry into chicken flocks. Antibiotics are commonly used to treat chicken flocks affected with colibacillosis.

APEC can affect all species of poultry in all types of production systems [3]. APEC is also prevalent (9.52% to 36.73%) in all age groups of chickens [7]. Broiler chickens between the ages of 4 and 6 weeks are more susceptible [1], whereas layer chickens can be affected by APEC throughout the grow and lay periods, particularly around the peak egg production and late lay period [1]. In the US, it is estimated that at least 30% of commercial flocks are affected by APEC at any point of time [8]. Multiple APEC serotypes have been associated with colibacillosis cases in the field outbreaks; however, three serotypes (O78, O2, and O1) account for the majority (more than 80%) of the cases [1,5]. APEC leads to systemic infections in chickens either as a primary pathogen or secondary to viral (infectious bronchitis (IBV), Newcastle disease (NDV), avian influenza (AIV)) and *Mycoplasma* (*Mycoplasma gallisepticum* (MG)) infections, immunosuppressive disease (infectious bursal disease (IBD)), or environmental stresses (overcrowding, high level of dust and ammonia) by entering through oral and respiratory routes [1,5]. Interestingly, studies have shown that APEC can colonize the gastrointestinal and respiratory tracts of chickens without causing disease and only translocate to extra-intestinal sites in the presence of stressors (production-related stress, immunosuppression, and concurrent infections) as an opportunistic pathogen [2,9]. APEC invades the gastrointestinal and respiratory tracts through abraded tracheal and intestinal epithelium in the presence of stressors and reaches bloodstream and internal organs [1,2,3]. Chickens get infected through contaminated feed and water and can spread to other birds through the feco-oral or aerosol route [1,2,3]. Furthermore, APEC can be vertically transmitted from infected breeders via contaminated eggs [1,2,3]. An overview of APEC infection in chickens is shown in Scheme 1.

APEC utilizes different virulence and pathogenesis factors to cause disease in chickens, primarily adhesins, invasins, protectins, iron acquisition systems, and toxins [2]. These factors facilitate adhesion, invasion, evasion from the host immune responses, colonization, proliferation, and systemic dissemination of APEC, thereby allowing the establishment of infection in chickens [2]. In addition to these factors, several other bacterial factors including but not limited to secretion systems (type III and VI), quorum sensing (QS) systems, transcriptional regulators, two-component systems, and metabolism-associated genes also contribute to APEC pathogenesis in chickens [10,11,12,13,14,15,16,17,18]. An in-depth understanding of these factors and their roles in APEC pathogenesis will help to develop new effective preventative and therapeutic treatments.

Recent studies suggest APEC (particularly isolates belonging to sequence types ST95 and ST131 or O1, O2, and O18 serogroups) as a potential foodborne zoonotic pathogen as well as a source or reservoir of extra-intestinal infections in humans [4,19,20,21]. Particularly, APEC shares genetic similarity with human ExPECs, uropathogenic *E*. *coli* (UPEC), and neonatal meningitis *E*. *coli* (NMEC) and possesses UPEC-and NMEC-defining virulence genes with the ability to cause urinary tract infections (UTI) and meningitis in mice and rat models [4,22]. Furthermore, the detection of APEC-specific ColV (colicin V) plasmids in human ExPEC isolates suggests a possible zoonotic transmission of APEC from poultry to humans [21]. Therefore, APEC is a pathogen of importance to the poultry industry and public health.

Antibiotics (tetracyclines, sulfonamides, and aminoglycosides) are frequently used to control colibacillosis in chickens [23]. However, increasing resistance of APEC to different classes of antibiotics, including medically important antibiotics (β-lactams, colistin, and carbapenems), suggests challenges ahead in using antibiotics to control APEC infections in chickens [24]. Furthermore, there is no effective vaccine available to protect chickens against APEC infections, which is mainly due to the diversity of APEC serotypes associated with colibacillosis cases in field outbreaks [5]. Currently, only two vaccines (live-attenuated APEC O78 Δ*aro*A Poulvac^®^ *E. coli* vaccine and inactivated Nobilis^®^ *E*. *coli* vaccine containing F11 fimbrial and FT flagellar antigens) are commercially available for use in chickens [5,25]. These scenarios necessitate the development of new and alternative therapies to control APEC infections in chickens. Probiotics, bacteriophages, and different new therapies (innate immune stimulants, growth and QS inhibitors, and antimicrobial peptides) have shown promising efficacy in reducing APEC infections in chickens [26,27,28,29,30]; however, none of these has advanced into field applications to date.

Here, we discuss the virulence and pathogenesis factors of APEC and their roles in systemic infections in chickens, review the zoonotic potential, provide the current status of antibiotic resistance in APEC and progress in vaccines development, and summarize the alternative control measures under investigation. The PubMed database was used for advanced search (PubMed Advanced Search Builder) of the articles using key words: “avian pathogenic *Escherichia coli*”, “virulence”, “pathogenesis”, “human infections”, “control”, “antibiotics”, “antimicrobials”, “resistance”, “vaccine”, “probiotics” and “phages”. Single key word “avian pathogenic *Escherichia coli*” and two key words consisting of “avian pathogenic *Escherichia coli*” and either of “virulence”, “pathogenesis”, “human infections”, “control”, “antibiotics”, “antimicrobials”, “resistance”, “vaccine”, “probiotics” or “phages” was used for each advanced search.

## 2. Virulence and Pathogenesis Factors

APEC possesses or utilizes different virulence and pathogenesis factors or mechanisms to cause colibacillosis in poultry [2,10,11,12,13,14,15,16,17,18,31]. These factors include but are not limited to adhesins, invasins, protectins, iron acquisition systems, toxins, two-component systems, a quorum-sensing (QS) system, transcriptional regulators, secretion systems, and genes associated with metabolism [2,10,11,12,13,14,15,16,17,18,31]. These factors play various roles in APEC infections, including attachment to host cells, invasion of the host cells, survival inside the phagocytic (macrophages) cells, colonization of tissues, persistence in the bloodstream, proliferation/replication inside the cells, cell lysis and damage, sequestering metals from body fluids for growth, resistance to serum bactericidal activity and oxidative and environmental stresses, motility, and biofilm formation [2,10,11,12,13,14,15,16,17,18,31]. Table 1 provides the list of virulence and pathogenesis factors defined or characterized in APEC to date along with their roles in APEC pathogenesis/infection.

### 2.1. Adhesins

Adhesins are appendages or cell-surface components of bacteria that facilitate adhesion or adherence to other cells or to surfaces, usually in the host they are living in or infecting [2,31]. Adherence is required for colonizing a new host and is an essential step in bacterial pathogenesis or infection [2,31]. Adherence in APEC is facilitated primarily by type 1 fimbriae, P fimbriae, and S fimbriae [2,31]. Several genes encoding these fimbriae and additional adhesins, *fim*H, *fim*C (type 1 fimbriae), *pap*A, *pap*C, *pap*EF, *pap*G I, *pap*G II, *pap*GIII, *fel*A (P fimbriae), *sfa*/*sfa*S (S fimbriae), *foc*GE (F1C fimbriae), *afa*IBC (afimbriae), *lpf*A, *lpf*0141, *lpf*0154 (long polar fimbriae), *mat*/*ecp*A (fimbrillin), *flg*E (flagellar hook), *crl*, *csg* (curli), *tsh* (temperature-sensitive haemagglutinin), *bma*E (M hemagglutinin), *hra*/*hrl*A/*hek* (heat-resistant agglutinin), *iha* (IrgA homologue adhesin), *yqi*G (putative outer membrane usher protein), and *kii* (K capsule encoding genes) have been reported in APEC [32,33,34,35,36,37,38,39,40,41]. These adhesins also mediate motility, biofilm formation, and APEC survival in macrophages [31]. Furthermore, the fimbriae-encoding gene, *yfc*O, facilitates adhesion, colonization, and resistance to environmental stresses [42], whereas *yad*C, promotes adhesion, intracellular survival, and motility [43]. Similarly, autotransporter adhesin genes (*aat*A, *aat*B, *upa*B) contribute to adhesion, colonization, and biofilm formation [44,45]. By screening a random transposon mutant library, multiple other genes (*fdt*A, *rlu*D, *yjh*B, *ecp*R, and *fde*C) were found to be responsible for adhesion to chicken and human cell lines [46].

### 2.2. Invasins

Invasins are a class of proteins associated with the entry of pathogens into host cells [2,31]. Invasins play a role in promoting entry during the initial stage of the infection [2,31]. Multiple genes encoding invasins, *ibe*A (also called *ibe*10), *ibe*B (invasion protein), *tia* (toxigenic invasion locus), and *gim*B (genetic island associated with neonatal meningitis) have been reported in APEC isolates [35,47,48]. In addition, invasins also contribute to APEC resistance to oxidative stress induced by macrophages, biofilm formation, colonization, and proliferation in the host [47,48]. IbeR, a regulator of *ibe*RAT operon, contributes to invasion, resistance to serum and environmental stresses, and the expression of virulence genes [49]. Similarly, *ych*O, a putative invasin gene, plays a role in motility, adhesion, invasion, biofilm formation, and the expression of membrane proteins and metabolism genes [50].

### 2.3. Iron Acquisition Systems

Iron is an essential micronutrient required for bacterial growth and proliferation inside the host, once bacteria successfully colonize and/or invade the host [2,31]. APEC possesses different iron acquisition systems consisting of multiple siderophores (aerobactin, salmochelin, yersiniabactin) and transporters to sequester iron from the body fluids [2,31]. Several genes encoding the iron uptake and transport systems, *iuc*CD, *iut*A, *aer*J (aerobactin), *iro*BCDEN (salmochelin), *fyu*A (yersiniabactin), *sit*ABCD, *mnt*H (iron and manganese transporter), *irp*2 (iron repressible protein), *feo*B (ferrous ion transporter), *fep*C (ferric enterobactin transporter), *ire*A (iron-regulated virulence gene), *eit*ABCD (putative iron transporter), *chu*A (outer membrane hemin receptor), and *bfr* (bacterioferritin) have been reported in APEC [36,37,40,51,52,53,54,55,56,57]. In addition, these siderophores and transporters also mediate APEC adhesion, invasion, resistance to environmental stresses, the expression of other virulence genes, colonization, and persistence in the host [52,56,58,59]. Furthermore, enterobactin synthesis and transport genes (*ent*E and *ent*S) in coordination with gene encoding outer membrane efflux protein (*tol*C) also facilitate invasion, colonization, and persistence [60].

### 2.4. Protectins

Protectins protect bacteria from the host immune system as well as various unfavorable conditions [2,31]. In particular, protectins include bacterial capsules, outer membrane proteins, and lipopolysaccharide (LPS) components, and they provide protection against phagocytic engulfment by macrophages and complement-mediated bactericidal effect in the host serum [2,31]. Several genes encoding multiple protectins, *iss* (increased serum survival), *tra*T (complement resistance protein), *omp*T (outer membrane protease), *kps*MT(K1), *kps*MT(II), *kps*MT(III), *neu*C, *neu*S, *neu*D (capsule), *kfi*C-K5 (glycosyl transferase), and *bet*A (choline dehydrogenase) have been reported in APEC [36,41,51,53,61]. These protectins also mediate APEC adhesion, invasion, intracellular survival, colonization, and proliferation in the host, in addition to protection from host defense [61]. The outer membrane proteins, YbjX and PagP, also play a role in resistance to serum and environmental stresses, invasion, and intracellular survival [62,63]. Similarly, OmpA, another outer membrane protein, also promotes APEC survival in macrophages [64]. The genes involved in LPS biosynthesis, *wzy* (O-antigen polymerase) and *waa*L (O-antigen ligase), facilitate intracellular survival and resistance to phagocytosis and environmental stresses along with adhesion, invasion, colonization, motility, and biofilm formation [65,66]. Similarly, *lpx*M (myristoyl transferase), a gene involved in lipid A biosynthesis, plays a role in invasion, intracellular survival, colonization, and regulation of cytokine genes expression and nitric oxide production [67]. Whereas, *sod*A (superoxide dismutase) protects APEC from reactive oxygen species (ROS)-mediated host defense and promotes biofilm formation [68].

### 2.5. Toxins

Toxins are biological poisons that assist in the bacterial ability to invade and cause damage to the tissues [2,31]. Several genes encoding multiple types of toxins, *hly*F, *hly*A, *hly*E (putative avian hemolysin), *vat* (vacuolating autotransporter toxin), *sat* (secreted autotransporter toxin), *cdt*B, *cdt*S (cytolethal distending factor), *ast*A, EAST-1 (heat-stable enterotoxin), stx2f (shiga toxin variant), *pic* (serine protease autotransporter), espC (serine protease), and *ace*4/35 (acetylcholine esterase) have been reported in APEC [35,38,39,40,41,51,53,69,70,71,72,73]. These toxins also facilitate the colonization, motility, biofilm formation, agglutination, induction of vacuolization, and formation of outer membrane vesicles [69].

### 2.6. Other Virulence and Pathogenesis Factors

Other virulence and pathogenesis factors of APEC include the QS system, transcriptional regulators, two-component systems, secretion systems, and genes associated with bacterial metabolism [10,11,12,13,14,15,16,17,18]. These factors assist in different processes of APEC pathogenesis/infection, including adhesion, invasion, colonization, persistence, interbacterial competitions, resistance to host defenses, and modulation of host immune responses [10,11,12,13,14,15,16,17,18], thereby facilitating the APEC proliferation and establishment of disease in the host.

#### 2.6.1. Quorum-Sensing (QS) System

Quorum sensing is an autoinducer (small hormone-like organic molecules)-based cell-to-cell communication system in bacteria that regulates the expression of various genes associated with motility, biofilm formation, virulence, and others [74]. QS in APEC is mediated by a LuxS synthesized autoinducer-2 (AI-2) molecule and regulated by LsrABCDFGK operon [10,74,75]. Lsr operon, LuxS, and AI-2 along with *pts*I (phosphotransferase system), and Pfs (activated methyl cycle pathway) play various roles in APEC pathogenesis, including motility, biofilm formation, adherence, invasion, colonization, intracellular survival, persistence, cell damage, and the expression of virulence genes [10,74,75,76,77].

#### 2.6.2. Secretion Systems

The secretion systems are cell-associated systems that are present on cell membranes of bacteria and function to secrete proteins into host cells, thereby causing damage to the host cells [11,12]. The secreted proteins promote the bacterial virulence either by directly intoxicating the host cells or by enhancing attachment to host cells, establishing replicative niche by scavenging resources and by competing with other microorganisms. Among the different bacterial secretion systems, two secretion systems (type III and VI) contribute to APEC pathogenesis [11,12]. The regulators (EtrA and YqeI) [12,78] and ATPase (EivC) [79] of the type III secretion system 2 (ETT2) play a role in motility, adhesion, intracellular survival, proliferation, colonization, resistance to phagocytosis and serum bactericidal activity, expression of fimbriae genes, and the downregulation of pro-inflammatory cytokine responses. Similarly, different components of type VI secretion system, DotU (organelle trafficking protein), IcmF (intracellular multiplication factor), Hcp (hemolysis co-regulation protein), CpxR, CpxA (envelope stress response system), ClpV (ATPase), and VrgG (secreted protein) mediate interbacterial competition, adhesion, invasion, intracellular survival, colonization, motility, biofilm formation, production of type 1 fimbriae, resistance to serum bactericidal activity, and modulation of intracellular host responses (IL-8, IL-1β) [11,40,80,81,82,83,84,85].

#### 2.6.3. Two-Component Systems

Two-component systems (TCS) are major signaling proteins in bacteria that enable bacteria to respond to changing environments by altering the expression of genes [86]. Different TCSs have been reported with a role in APEC pathogenesis [86,87,88,89]. A membrane-associated TCS, PhoPQ, plays a role in biofilm formation, motility, adhesion, invasion, intracellular survival, systemic dissemination, and the expression of virulence genes and genes associated with flagellar assembly, ABC transporters, quorum sensing, and bacterial chemotaxis [13,86,90]. Similarly, another membrane-associated TCS, BasSR, is involved in biofilm formation and APEC virulence and colonization in vivo [91]. KdpDE, a TCS regulating potassium transport, mediates the expression of flagella-related genes, flagellum formation, motility, and resistance to serum bactericidal activity [87]. Likewise, a TCS regulating nitrogen metabolism, RstAB, contributes to iron acquisition, acid resistance, intracellular survival, and colonization [88,92]. Another TCS, BarA-UvrY, plays a role in the adhesion, invasion, persistence, intracellular survival, resistance to serum bactericidal activity and oxidative stress, and regulation of exopolysaccharide production and expression of type 1 and P fimbriae [89].

#### 2.6.4. Transcriptional Regulators

Multiple transcriptional regulators have shown a role in APEC pathogenesis [14,15,37,93,94,95]. The AutA and AutR, two global transcriptional regulators, mediate the expression of K1 capsule and acid resistance systems, and change in adaptive lifestyle to facilitate infection [14]. FNR (fumarate and nitrate reduction), another global transcriptional regulator, facilitates the adhesion, invasion, expression of type 1 fimbriae and type VI secretion system, and resistance to oxidative stress [15]. McbR (MqsR-controlled colonic acid and biofilm regulator) plays a role in biofilm formation and stress response [94], whereas *tyr*R (a transcriptional regulator involved in the biosynthesis and transport of aromatic amino acids) promotes invasion, motility, and intracellular survival [37]. YjjQ (transcriptional regulator, LuxR family) contributes to flagellar motility [93], and RfaH, a transcriptional anti-terminator, contributes to invasion, intracellular survival, and resistance to serum bactericidal activity [95].

#### 2.6.5. Metabolism-Associated Genes

Different genes associated with bacterial metabolism contribute to APEC pathogenesis [16,17,18,96]. The operon, *acs-yjcH-actP*, encoding acetate assimilation system facilitates intracellular survival, proliferation, colonization, and the production of pro-inflammatory cytokines and nitric oxide [96]. Similarly, PotE (putrescine transporter) and NirC (nitrite transporter), involved in polyamine biosynthesis and putrescine transport, and nitrogen metabolism and cytoplasmic detoxification, respectively, mediate adhesion and colonization [16,17]. ArcA (aerobic respiratory control), involved in citrate transport and metabolism, plays a role in motility and chemotaxis [18].

#### 2.6.6. Miscellaneous

Various other bacterial components, such as genes encoding prophage, porins, enzymes, hypothetical protein, and transport systems also play a role in APEC pathogenesis. Prophage phiv142-3, particularly *orf*20 gene, and phiv205-1 contributes to resistance to serum and environmental stresses, adhesion, invasion, intracellular survival, colonization, biofilm formation, and formation of flagella and type 1 fimbriae [97,98,99]. Outer membrane porins, OmpF and OmpC, facilitate adhesion, invasion, colonization, and proliferation [100]. The hypothetical protein, YicS, plays a role in motility, biofilm formation, and invasion [101]. While, *cpd*B (2′, 3′-cyclic phosphodiesterase) mediates colonization [102], the phosphate transport system (*pst*SCAB), particularly *pst*B, plays a role in colonization as well as resistance to serum bactericidal activity and oxidative stress [103]. The transfer-mRNA-small protein B, tmRNA-SmpB, mediates colonization, persistence, replication, and intracellular survival [104], and *mli*C, a lysozyme inhibitor, plays a role in resistance to serum bactericidal activity [105]. Additionally, different virulence genes of unknown/not clearly known functions in APEC (but defined in other bacteria), *mal*X (enzyme II of phosphotransferase system recognizing glucose and maltose) [53], *ets*B (putative ABC transport system) [54], *uid*A (beta-glucuronidase), *usp* (uropathogenic specific protein), and *hem*F (oxygen-dependent coproporphyrinogen-III oxidase) have been also reported. *mal*X plays a role in sugar transport, whereas *ets*B is type VI secretion system-associated gene. *uid*A, *usp*, and *hem*F encode beta-glucuronidase that break down carbohydrates, non-specific nuclease that cleaves nucleic acids, and coproporphyrinogen-III oxidase involved in heme biosynthesis, respectivley. Furthermore, other genes such as HJ *fli*C (flagellin), *clb*B, *clb*N (colibactin) [39,40], *frz* (carbohydrate metabolic operon), *sop*B (plasmid partitioning protein) [106], *cva*ABC, *cvi* (colicin V operon) [34], *cib*/*cib*I (colicin Ib), *cbi* (colicin M immunity protein) [57], *cba* (colicin B activity protein), and *cma* (colicin M activity protein) have been also reported. *fli*C encodes flagellin essential for flagellar motility, whereas *clb*B and *clb*N are colibactin genes involved in fatty acid biosynthesis. Colicin genes (*cva*ABC, *cvi*, *cib*/*cib*I, *cbi*, *cba*, and *cma*) are associated with bacteriocin production to exert cytotoxic effects against other bacteria in the niche. *frz* plays a role in sugar (fructose) transport, whereas *sop*B is associated with plasmid replication. Moreover, genes such as *yfc*V (fimbrial protein), *gad* (glutamate decarboxylase), *mch*BCF (microcin H47), *mcm*A(microcin M), *bor* (bacteriophage lambda bor protein), *air* (enteroaggregative immunoglobulin repeat protein), *eil*A (homolog of transcriptional regulator *hil*A) [107], *cel*B (permease IIC component), and *pab*B (aminodeoxychorismate synthase) [40] have been also reported. *yfc*V encodes uncharacterized fimbrial-like protein, whereas *gad* is involved in acid tolerance. Microcin genes (*mch*BCF and *mcm*A) are associated with antibacterial activity against closely related species. *bor* is involved in serum resistance, whereas *air* encodes protease facilitating mucin resistance. *eil*A is a component of a type III secretion system, whereas *cel*B and *pab*B are involved in sugar transport and tetrahydrofolate biosynthesis, respectively. In addition, genes such as *cap*U (cap locus protein), *cif* (cell cycle inhibiting factor), *tir* (translocated intimin receptor), *tccp* (tir cytoskeleton coupling protein), *nle*B (non-LEE encoded effector B) [57], *ia*L (invasion-associated locus), *cjr*C (putative siderophore receptor) [108], and *mig-14p* (antimicrobial resistance protein) [41] have been also reported in APEC. *cap*U is putative hexosyltransferase with uncharacterized function, whereas *cif* belongs to bacterial toxins that arrest host cell division. *tir* and *tccp* are involved in bacterial adherence to host cells, whereas *nle*B is a component of a type III secretion system. *ia*L is involved in cell penetration, whereas *cjr*C is a putative siderophore receptor. *mig-14p* plays a role in resistance to antimicrobial peptides in host cells.

### 2.7. Genes Essential for Systemic Infections and Adaptation in Chickens

Identifying the genes essential for systemic infections and adaptation is crucial to develop rational treatments against the infections. Nelwike et al. (2012) [109] investigated the APEC genes induced during systemic infections in chickens using RIVET (recombination-based in vivo expression technology). Genes involved in metabolism, cell envelope and integrity, transport systems, and virulence (*met*H, *lys*A, *pnt*A, *pur*L, *ser*S, *ybj*E, *ycd*K (*rut*C), *wca*J, *gsp*L, *sds*R, *irp*2, *eit*D, *ylb*E, *yji*Y, *tkt*1, and phage-related genes) were upregulated in APEC isolated from infected chickens. Similarly, Dozois et al. (2003) [110] studied the APEC genes expressed in infected tissues in chickens using SCOTS (selective capture of transcribed sequences) technology. Genes involved in adherence (*pil*N, *pil*Q, *tsh*, *hpb*, TcfD, Z5222), LPS synthesis (*waa*O, *waa*Y), iron acquisition (*iut*A, *iuc*A, *iuc*D, *iro*C), plasmid function (ColE2, *tra*K, *tra*G, *tra*T, *Sop*A, *psi*A), phage-related (*hka*G, *hkb*V, *hkb*Q, Z3370, Int), and of unknown functions (CC0532, TM0427, YPO3000, *rhs*H, RSp0733) were highly expressed in APEC infected tissues. On the other hand, Zhang et al. (2019) [111] identified essential genes for APEC adaptation in chickens using the TraDIS (transposon-directed insertion site sequencing) strategy. Genes involved in metabolism, transport, regulation and stress response, RNA processing and translation, cell division and DNA replication, cell envelope biogenesis, and unknown functions were essential to cause disease in chickens. Particularly, genes involved in biotin synthesis (*bio*ABFCD), Rnf electron transport complex (*rnf*A, *rfn*E, and gene *nth* encoding endonuclease III), and cre two-component system (*cre*ABCD) were important for the adaptation of APEC in chickens. In another study, genes identified as upregulated through microarray analysis (*yeh*D, *pot*F, *flg*E, *tyr*R, and *bfr*) in APEC isolated from chicken showing swollen head syndrome were essential for adhesion, invasion, survival inside macrophages, and motility [37]. These studies provide insights on APEC pathophysiological processes during systemic infections in chickens.

Overall, multiple virulence and pathogenesis factors of APEC are involved in causing colibacillosis in poultry. As a result of the involvement of multiple virulence and pathogenesis factors, there is a hindrance in developing therapeutics broadly effective against APEC infections. In-depth understanding of these factors as well as unraveling the new factors will help develop the effective therapeutics against colibacillosis in poultry. Furthermore, several of these factors have coordinated and overlapping functions, which necessitates a holistic strategy to formulate an ideal anti-APEC therapeutics. For instance, developing therapeutics targeting iron acquisition systems [112], QS system [113], bacterial metabolism [114], and secretion systems [115] can provide solutions to mitigate APEC infections in poultry in the future.

## 3. Zoonotic Potential

APEC belongs to the ExPEC subgroup of *E*. *coli*, similar to UPEC and NMEC [4]. Multiple studies have reported APEC as a potential foodborne zoonotic pathogen as well as a source or reservoir of extra-intestinal infections in humans [21,22,41,116,117,118,119,120,121,122,123,124,125,126,127,128,129,130,131,132,133,134,135,136,137,138,139,140,141,142,143]. This is in particular due to its shared genetic similarity with human ExPECs, the presence of common or human ExPEC-defining virulence genes, and the ability to cause UTI and meningitis in rodent models, similar to UPEC and NMEC.

### 3.1. Genetic Similarity and Commonality in Virulence Genes

Multiple studies have shown that APEC shares genetic similarities with human ExPECs (UPEC and NMEC) and avian-associated ColV plasmids (for instance, pAPEC-078-ColV, pAPEC-O2-ColV, pAPEC-O1, p1ColV5155) essential for poultry adaptation are present in human ExPEC isolates. The phylogenetic (single nucleotide polymorphisms; SNP) comparison of whole-genomes of 323 APEC and human ExPEC isolates belonging to sequence type ST95 revealed genetic overlap (no distinct clustering) between APEC and certain human ExPECs, indicating that certain ExPEC clones may have the potential to cause infection in both poultry and humans [116]. Especially, ColV plasmids specific to APEC were present in those human ExPEC isolates, and 10 virulence genes (*iuc*C, *iuc*D, *iut*A, *cva*A, *ets*A, *hly*F, *omp*T, *cva*B, *cva*C, and *cvi*) were common between them. Another study compared the whole genomes of 48 APEC and UPEC isolates belonging to ST131-H22 [21]. The high genetic similarity (no distinct lineage) was observed in SNP-based phylogenetic analysis between UPEC and APEC isolates together with the presence of ColV plasmids in UPEC isolates. The ColV/ColBM plasmids were also present in 34.5% of APEC and 18.9% of human ExPEC isolates when whole genomes of 551 *mcr*-1 positive APEC and human ExPEC isolates were analyzed [118]. Therefore, the presence of poultry-specific ColV plasmids in human ExPEC isolates might suggest a zoonotic transmission of APEC from poultry to humans.

Other studies have also shown the commonality in virulence genes between APEC and human ExPEC isolates. Four virulence genes (*iss*, *iut*A, *omp*T, and *pap*GII) were common when the presence of virulence genes was investigated in 200 UPEC and APEC isolates [117]. In another study, several virulence genes (*iro*N, *tra*T, *iuc*D, *cvi*/*cva*, *ibe*A, *gim*B, *tia*, *neu*C, *kps*MTII, *tsh*, *iss*, *sit*D, *chu*A, *fyu*A, *irp*2, *vat*, *mal*X, and *pic*) were present in APEC, UPEC, and NMEC isolates [123]. The screening of virulence genes in 27 APEC isolates belonging to ST73 found different virulence genes (*pap*, *sfa*, *usp*, *cnf*1, *kps*MTII, *hly*A, and *ibe*A) known to be specific to UPEC and NMEC [119]. The human ExPEC-defining virulence markers (*pap*AH, *pap*C, *pap*EF, *pap*G, *pap*G allele II, *fyu*A, *kps*MTII, K1, *usp*, *ibe*A, and *iut*A) were also highly prevalent in 25 APEC isolates belonging to ST95, ST127, ST131, ST141, ST420, and ST69 [120]. Similarly, different human ExPEC-defining virulence markers (*pap*A, *pap*C, *pap*EF, *pap*G, *hra*, *ast*A, *hly*F, *tsh*, *fyu*A, *ire*A, *iro*N, *iut*A, *cva*C, *iss*, *kps*MT K1, kii, *tra*T, *ibe*A, *omp*T, *mal*X, *usp*, and *fim*H) were prevalent in 129 ExPEC isolates from meat and shell eggs [121]. Four human ExPEC-defining virulence markers (*pap*A, *pap*C, *kps*MII, and *iut*A) were also prevalent in APEC isolates from broiler chickens and meat [122]. Virulence determinants/markers associated with UPEC (growth in human urine, *iha*, *foc*, *cnf*, *pap*C, *pap*GII, *iro*N, *iut*A, *kps*MTII, *cva*C, *hly*F, *vat*, *mal*X, *usp*) and NMEC (*kps*MTK1 and *ibe*A) were present in ExPEC isolates from chicken samples, including feces and cecal contents [143]. Therefore, the presence of common virulence traits between APEC and human ExPEC isolates can provide substantial evidence of zoonotic threats posed by APEC to humans.

Several other phylogenetic studies have also shown the similarity of APEC with human ExPEC isolates. The phylogenomic tree constructed based on whole genomes of 47 *E*. *coli* strains revealed that APEC isolates belonging to serogroups O1:K1 and O2:K1 share significant genetic similarities/overlap (same cluster) with human ExPEC O18:K1 strains [124]. This finding is further supported by several other studies [22,125,126,127,128,129,136]. The ExPEC (APEC, UPEC, and NMEC) isolates belonging to ST95 and serogroups O1, O2, and O18 were clustered together when the phylogeny of ExPEC isolates was constructed based on the possession of different genes/traits or multilocus sequence typing (MLST), suggesting the potential transmission of certain ExPEC strains between poultry and humans [125,127]. Similarly, APEC isolates belonging to serogroup O18 were similar to NMEC strains when compared using MLST and pulsed-field gel electrophoresis (PFGE) [22,129]. In other studies, APEC isolates belonging to ST95 and serogroup O1 were also similar to UPEC and NMEC isolates when compared using MLST, PFGE, and whole genome analysis, suggesting the zoonotic potential of these isolates to humans with no host-specificity [126,128]. In several other studies performed using MLST and PFGE, APEC isolates belonging to sequence type other than ST95, such as ST359 [130], ST23 [131], ST10, ST117, ST746 [132], ST617, ST23 [135], or other serogroups such as O45 [133,134] were also similar to human ExPEC isolates. Therefore, the APEC isolates belonging to certain STs or serogroups could pose a significant zoonotic risk to humans.

### 3.2. Ability to Cause Disease with Similar Clinical Manifestations

Multiple studies have shown that APEC can cause UTI and meningitis similar to UPEC and NMEC, respectively [22,124,137,138,139,140]. The *E. coli* isolates from chicken meat and shell eggs were lethal similar to UPEC in a mouse model of UTI, caused sepsis in a mouse sepsis model, and infected the cerebrospinal fluid (CSF) similar to NMEC in a neonatal rat meningitis model [137]. Furthermore, these isolates also possessed swimming (motility) and biofilm-forming ability in urine, and they adhere, invade, and survive intracellularly in human kidney and bladder cells, similar to UPEC. These isolates also possessed K1 capsule and *ibe*A, which are essential virulence factors for NMEC pathogenesis. Similar findings were also observed in other studies [22,41,124,138,139,140]. The *E. coli* isolates from chicken feces or from colibacillosis lesions were also lethal, caused sepsis, and infected CSF in rodent model studies [41,138]. In another study, *E*. *coli* isolated from colibacillosis cases and belonging to phylogroup F were able to cause disease (sepsis, meningitis, and UTI) in animal models of human infections [41]. The APEC and ExPEC (UPEC and NMEC) isolates, belonging to ST95 when compared, both were equally able to adhere and invade kidney cells, form strong biofilm, and resist the bactericidal activity of serum [139], reinforcing the understanding that APEC isolates belonging to ST95 pose a potential zoonotic risk to humans. Other similar studies have found that APEC isolates, particularly belonging to serogroup O18, survive in human serum, and they bind and enter human macrophages and human cerebral microvascular endothelial cells, similar to NMEC [22,140]. These isolates also induce neuronal apoptosis in mice, suggesting that APEC O18 isolates utilize similar pathogenic mechanisms as NMEC to cause meningitis in mice. Furthermore, APEC isolates, belonging to serogroup O1:K1 and O2:K1, also cause sepsis and meningitis in rodent models, suggesting that APEC O1:K1 and O2:K1 can have zoonotic potential [124].

Conversely, UPEC isolates also induced colibacillosis in chickens [141,142]. The experimental infection of laying hens with UPEC isolate caused salphingitis similar to APEC [141]. UPEC also induced similar symptoms and lesions comparable to those caused by APEC in chickens [144]. In another study, UPEC isolates belonging to serogroups O4, O74, O1, and O75 were 100% lethal to chickens [142]. The NMEC isolates were also lethal to chick embryos and caused colisepticemia in chickens, similar to APEC [22].

Overall, the potential of APEC ST95 and ST131 strains to cause UTI and meningitis in humans through the consumption of contaminated poultry products signifies the zoonotic nature of APEC. Furthermore, the zoonotic potential of APEC isolates, especially belonging to serogroups O1, O2, and O18, cannot be underestimated; therefore, interventions appropriate to mitigate the transmission of APEC to humans should be undertaken to combat the food safety threat posed by APEC to humans. Future investigations should consider determining the link between APEC and potential zoonotic transmission to humans.

## 4. Control Strategies

The control of APEC infections in poultry relies on antibiotic medication and vaccination, other than managing the environmental stressors, applying the biosecurity measures, and vaccinating the chickens against the viral and immunosuppressive diseases [1,2,145]. Probiotics, bacteriophages, and different new alternatives (innate immune stimulants, virulence and growth inhibitors, and antimicrobial peptides) have been also evaluated [26,27,28,29,30] with a goal to develop effective preventative and therapeutic treatments to control colibacillosis in chickens. Potential checkpoints for controlling APEC infection in chickens are shown in Scheme 1.

### 4.1. Management and Biosecurity Measures

The management of environmental stressors such as ammonia and dust in poultry houses by maintaining good litter and air quality are some of the key factors in preventing APEC infections in poultry houses [1,2]. Proper ventilation as well as maintaining optimum temperature, humidity, and bird density help mitigate environmental stress in chickens [1,2]. Furthermore, the elimination of pre-disposing factors by vaccinating chickens against MG, IBV, NDV, and IBD reduces the incidence of APEC infections [1,2]. Good nutrition and birds with enhanced immune systems are also likely contributors to reducing the incidence of colibacillosis [1,2]. Moreover, the vertical transmission of APEC can be prevented at the breeding level or at the top of the production pyramid by different intervention measures such as developing breeds with increased resistance to APEC infections, cleaning and disinfection of hatching eggs, and minimizing the use of floor eggs [145]. The horizontal transmission of APEC can be limited by using all-in-all-out production systems, systematic culling of weak chicks at first week, and implementing effective sanitation programs [145]. The proper and efficient biosecurity measures together with feed and water (chlorination) decontamination and disinfection of poultry houses, feed mills, farm equipment, and premises are necessary to prevent APEC entry into farms [1,2]. The biosafety measures such as preventing access of vectors such as houseflies, wild birds, and rodents are also necessary to keep APEC out of poultry facilities [145].

### 4.2. Antibiotics

Antibiotics are commonly used to control APEC infections in poultry [23]. Many antibiotics belonging to different classes, such as tetracyclines (tetracycline, oxytetracycline, chlortetracycline), sulfonamides (sulfadimethoxine, trimethoprim, sulfadiazine, sulfamethazine, sulfaquinoxaline, ormethoprim), aminoglycosides (apramycin, gentamicin, neomycin, spectinomycin), penicillins (amoxicillin, ampicillin), cephalosporins (ceftiofur), quinolones (danofloxacin, sarafloxacin, enrofloxacin), polymyxins (colistin), chloramphenicols (florfenicol), macrolides (erythromycin), and lincosamides (lincomycin) have been used in poultry industry worldwide for the control of APEC infections [23]. However, APEC resistance to multiple antibiotics has been reported [32,34,35,51,53,54,70,71,72,73,107,108,108,146,147,148,149,150,151,152,153,154,155,156,157,158,159,160,161,162,163,164,165,166,167,168,169,170,171,172], which limits the use of these antibiotics and suggests a challenge ahead in using these antibiotics. Table 2 provides a summary of antibiotic resistance and resistance genes (mechanisms) reported worldwide in APEC isolates from chickens in the last five years (2015 to 2020). These data indicate APEC resistance to almost all classes of antibiotics, except carbapenems. Resistance to imipenem has been also recently reported [108,108,171]. The resistance is most commonly seen with ampicillin, tetracycline, trimethoprim, sulfamethoxazole, and streptomycin antibiotics. Importantly, a high level of APEC resistance to medically important antibiotics, such as β-lactams and colistin, have also been reported, which might pose a high risk to humans because of the transmission of antibiotic-resistant bacteria and genes through the food chain [173]. The strategies employed by the US and European Union (EU) to restrict the non-therapeutic use (for growth promotion) of antibiotics in food–animal production and to limit the therapeutic use (for treatment) of medically important antibiotics could aid in mitigating this risk [174]; however, benefits of such measures in curbing antibiotic resistance issues may take time to realize. The development of antibacterials solely for animal uses without cross-resistance potential or the identification of antibiotic alternatives as a replacement for antibiotics could aid in combating antibiotic resistance issues in the food–animal production.

### 4.3. Vaccines

Various vaccine candidates, mostly live-attenuated and recombinant vaccines, have been investigated to protect chickens against APEC infections [5,175,176,177,178,179,180,181,182,183,184,185,186,187,188,189,190,191,192,193,194,195,196,197,198,199,200,201,202,203,204,205,206]. Table 3 provides the summary of vaccines tested to date along with their main findings. In the past, inactivated vaccines were tested; however, recent studies have focused mostly evaluating live-attenuated and recombinant vaccines in chickens. The varying degrees of protection, ranging from none to partial to complete, have been achieved using these vaccines. Among the tested vaccines, multiple vaccines such as outer membrane vesicles (OMVs), bacterial ghost vaccines, recombinant *iss*, recombinant antigen (rAg) vaccine containing ExPEC antigens, *Salmonella*-delivered vaccines containing APEC antigens, Δ*aro*A, and Δ*ton*B/Δ*fur* were able to reduce the mortality, lesions, and bacterial burden as well as stimulate the antibody (immunoglobulins; IgG and IgA) responses in chickens.

Despite multiple vaccine candidates showing proven efficacy in chickens in experimental studies, only two vaccines (live-attenuated APEC O78 ΔaroA Poulvac^®^ *E. coli* vaccine and inactivated Nobilis^®^ *E*. *coli* vaccine containing F11 fimbrial and FT flagellar antigens) are commercially available currently for use in chickens [5]. However, the major drawback of these vaccines is the lack of protection against heterogenous APEC infections [5]. The ideal APEC vaccine should be able to confer cross-protection against multiple APEC serotypes and be deliverable by mass-immunization methods, such as oral (feed or water) or spray routes [5]. The identification of common/conserved virulence and pathogenesis mechanisms employed by diverse APEC serotypes to cause disease in chickens would facilitate the development of new broad-spectrum vaccines. For instance, vaccine developed using outer membrane iron receptors required for iron acquisition (FyuA, Hma, IreA, IutA) protects against UPEC infections in a murine model [112]. In another study, a tetravalent conjugate vaccine developed using O antigens of predominant UPEC serotypes provides broad protection against UPEC infections [207]. Furthermore, the knowledge gained on new virulence and pathogenesis factors should be exploited to design potent vaccine candidates. For example, the type VI secretion system (*vgr*G) and quorum sensing system (*lux*S) can be the new vaccine targets because of their substantial involvement in APEC virulence and pathogenesis.

### 4.4. Probiotics

Different probiotics have been tested for their efficacy to prevent APEC infections in chickens [26,175,208,209]. The efficacy of *Lactobacillus plantarum* B1 was evaluated against *E. coli* (K88) infection by supplementing in the broilers feed (2 × 10^9^ CFU/kg) [26]. Broilers fed with *L. plantarum* B1 showed significantly decreased cecal *E. coli* counts and increased growth performance, villus height to crypt depth ratio, ileal mucosal sIgA concentration, and cecal lactic acid bacteria counts. Similarly, the efficacy of *L. plantarum* 15-1 and fructooligosaccharides (FOS) combination was evaluated against APEC (O78) infection by supplementing in the broilers feed (1 × 10^8^ CFU/kg) [208]. The decrease in mortality and serum diamine oxidase and increase in IgA and IgG concentrations was observed in broilers fed with probiotic and FOS mix. The effects of *Enterococcus faecalis*-1 was assessed in broiler chickens challenged with APEC (O78) by inoculating orally in drinking water for 3 days (1 × 10^8^ CFU) from days 1 to 3 of growth [210]. *E*. *faecalis*-1 supplementation significantly improved the growth performance and immune response, reduced the mortality, and decreased the visceral organs invasion by APEC O78. Likewise, the efficacy of multi-strain commercial probiotic mix (*Bacillus subtilis*, *Clostridium butyricum*, and *L. plantarum*) was tested against APEC 078 infection by supplementing in the broilers feed [209]. There was significant decrease in mortality (13.6% to 0%) and APEC counts in liver and spleen and increase in growth performance and lactobacilli population in broilers fed with the probiotic mix. Another commercial probiotic mix (*B. subtilis*, *L. acidophilus*, *Pediococcus acidilactici*, *Pediococcus pentosaceus*, and *Saccharomyces pastorianus*) was also tested in combination with recombinant attenuated *Salmonella* vaccine (RASV) for protection against APEC (O78:K80) and *Salmonella* infection by supplementing in feed in layer chickens [175]. Chickens showed reduced signs of airsacculitis, perihepatitis, and pericarditis and lower APEC load in the blood. These studies suggest that different probiotics belonging to genus *Lactobacillus*, *Bacillus*, *Clostridium*, and *Pediococcus* show efficacy in preventing APEC infections as well as improve the growth performance, maintain the healthy intestinal microbiota, and enhance the intestinal mucosal immunity. Furthermore, there are multiple probiotic products (for example, Sav-A-Chick^®^ Probiotic Poultry Supplement, Probios^®^, HealthyGutTM PROBIOTICS, and SuperDFM- Poultry) commercially available for use in maintaining intestinal health and boosting immune status in poultry. These probiotics contain different beneficial microorganisms, such as *B. subtilis*, *B. licheniformis*, *L. plantarum*, *L. casei*, *L. acidophilus*, *L. brevis*, *L. reuteri*, *Enterococcus faecium*, *E. thermophilus*, *P. acidilactici*, *P. pentosaceus*, *Bifidobacterium bifidum*, *B. animalis*, *Propionibacterium shermanii*, and *P. freudenerichii*. Even though these probiotics are not indicated specifically for APEC, they can reduce the incidence of APEC infections in poultry farms due to their broad-spectrum effect against enteric pathogens. Furthermore, the identification of new probiotics with superior potential to protect against APEC infections is necessary, which can provide alternatives to antibiotics, thereby also mitigating the development of antibiotic resistance. For instance, the next-generation probiotics specific to APEC can be developed by investigating the microbiome of healthy and APEC infected chickens followed by the identification of beneficial bacteria crucial to resist APEC infection in chickens [211].

### 4.5. Bacteriophages

To date, multiple studies have been conducted to evaluate the preventative and therapeutic efficacy of phages against APEC infections in chickens [27,212,213,214]. The efficacy of phage mixture (SPR02 and DAF6) was evaluated in APEC (O2) challenged chickens by spray and intramuscular administrations [214]. The phage treatment three days prior to APEC challenge significantly reduced (40% to 3%) the mortality of chickens. Similarly, phage treatment at 24 h and 48 h post-challenge also reduced the mortality rate (55% to below 20%). The efficacy of phage cocktail (phi F78E Myoviridiae, phi F258E Siphoviridae, and phi F61E Myoviridae) was tested in experimentally (O78) and naturally infected flocks refractive to antibiotic treatment by oral or spray administration [213]. The treatment with phage cocktail reduced mortality by 25% in experimentally infected chickens and decreased the flocks’ mortality level to below 0.5% in flocks infected naturally with APEC. Similarly, the efficacy of another phage cocktail (TM1, TM2, TM3, and TM4) was evaluated in APEC challenged (O78:K80 and O2:K1) chickens by administering through intramuscular injection [212]. The phage cocktail treatment reduced the mortality (46.6% to 13.6%), APEC load in lung, and APEC lesions in lung, liver, and heart, and increased the body weight of chickens. The efficacy of phage-loaded chitosan nanoparticles (C-ΦKAZ14 NPs; Myxoviridae, T4-like coliphage) was also evaluated in APEC-challenged (O1:K1:H7) chickens by oral administration [27]. The C-ΦKAZ14 NP treatment decreased the mortality (58.33% to 16.7%), intestinal colonization of APEC (2.30 × 10^9^ ± 0.02 to 0.79 × 10^3^ ± 0.10 CFU/mL), and fecal shedding (2.35 × 10^9^ ± 0.05 to 1.58 × 10^3^ ± 0.06 CFU/mL). C-ΦKAZ14 NP treatment also increased the body weight of chickens as well as ameliorated the clinical signs and symptoms. These studies suggest that the phage therapy can be a valuable approach to control APEC infections in chickens. However, no treatment involving phages has yet advanced into field applications. This is partly due to challenges in large-scale production and controversies associated with approval for use in poultry production [215]. If proven safe and effective in field settings, phages can serve as a valuable alternative to antibiotics in poultry production.

### 4.6. New Alternative Approaches

Apart from antibiotics, vaccines, probiotics, and bacteriophages, different novel approaches, including but not limited to innate immune stimulants, virulence and growth inhibitors, and antimicrobial peptides have been studied for their protective effects against APEC infections in chickens [28,29,216,217,218,219,220,221,222].

#### 4.6.1. Innate Immune Stimulants

Innate immune stimulants can activate the innate immune responses by acting as pathogen-associated molecular patterns (PAMPs) and binding to pattern recognition receptors (PRRs); thereby, preventing the host from infection [217]. The synthetic oligodeoxynucleotides containing unmethylated cytosine-phosphodiester-guanine motifs (CpG-ODN) was tested to protect neonatal chickens against APEC (O2) infection by administering through the intrapulmonary route using compressor nebulizer in an acrylic chamber [217]. Higher survival, better clinical conditions, and lower bacterial load were observed in chickens that received CpG-ODN. Furthermore, CpG-ODN also induced systemic antibacterial immune responses, including upregulation of expression of pro-inflammatory (IL-1β, LPS-induced tumor necrosis factor, IL-18) and anti-inflammatory (IL-10, IL-4) cytokine genes, and enrichment and maturation (higher CD40 and MHCII expression) of monocytes/macrophages and CD4^+^ and CD8^+^ T-cell subsets. Similarly, the prophylactic potential of three in ovo-administered innate immune stimulants and immune adjuvants (CpG-ODN, polyinosinic:polycytidic acid, and polyphosphazene) was evaluated to protect young chickens from yolk sac infection caused by APEC (O2) [28]. CpG-ODN increased the survival (>80%) of young chickens. Overall, these studies demonstrate that CpG-ODN can induce protective immunity against APEC-induced yolk sac infection in neonatal chickens. The evaluation in adult chickens to prevent APEC-associated infections is warranted to develop innate immune stimulants as new non-antibiotic measures for colibacillosis prevention.

#### 4.6.2. APEC Virulence and Growth Inhibitors

Virulence inhibitors disarm/attenuate pathogens by inhibiting virulence mechanisms, such as the QS system, unlike the antibiotics that inhibit the bacterial growth [223]. Therefore, virulence inhibitors can overcome the limitations of current antibiotics, viz. resistance and killing of commensal bacteria, and making pathogens susceptible to natural host defenses; thus, they are superior to conventional antibiotics [223]. On the other hand, growth inhibitors possessing novel scaffolds with newer antibacterial targets with less likelihood for resistance development such as those targeting bacterial membrane can be promising new antibacterial agents [224].

The protective effect of baicalin (medicinal ingredient isolated from dry roots of *Scutellaria baicalensis*), an APEC QS inhibitor, was evaluated against APEC-induced acute lung injury (O78) in chickens [218]. The pre-treatment of baicalin (200 mg/kg) significantly reduced the mortality, lesion in lung, lung wet/dry ratio, myeloperoxidase activity, and levels of pro-inflammatory cytokines (IL-1β, TNF-α, and IL-6) in the lung. In another study, the effect of rutin (flavonoid extracted from plants), an APEC QS inhibitor, was investigated on AI-2 secretion (APEC O78), biofilm formation, and the expression of virulence genes [29]. Rutin significantly reduced the AI-2 secretion, biofilm formation, and expression of virulence genes together with a decrease in adhesion and damage to chicken type II pneumocytes. Multiple APEC small molecule QS inhibitors, mostly piperazines, effective in reducing AI-2 secretion, biofilm formation, motility, the expression of virulence genes, and intracellular survival in macrophage and epithelial cells were identified through screening of the small molecule (SM) library [222]. The treatment with these QS inhibitors also increased the survival of wax moth (*Galleria mellonella*) larvae and decreased the intra-larval APEC load. Among the identified QS inhibitors, two QS inhibitors [QSI-5 (C-5) and QSI-10 (C-10)] [222] significantly reduced the APEC-induced mortality, lesions, and APEC load in internal organs of chickens (data not published). The effect of andrographolide (*Andrographis paniculata*) in reducing cell damage caused by APEC O78 was investigated in chicken type II pneumocytes [220]. Andrographolide significantly reduced AI-2 secretion, expression of virulence genes, the release of lactate dehydrogenase (LDH), F-actin cytoskeleton polymerization, and adhesion to chicken type II pneumocytes.

Multiple APEC small molecule growth inhibitors possessing pyrrolidinyl, imidazole, piperidine, quinoline, and nitrophenyl scaffolds were identified in our study through screening of the SM library [221,225]. These inhibitors were bactericidal to APEC, at very low concentrations, affected APEC membrane integrity, and decreased the intracellular survival of APEC in macrophage and epithelial cells. The treatment with these inhibitors also increased the survival of wax moth larvae and decreased the intra-larval APEC load. Among the identified growth inhibitors, two growth inhibitors [GI-7 (SM7) and GI-10 (SM10)] [221] significantly reduced the APEC-induced mortality, lesions, and APEC load in internal organs of chickens (data not published).

Altogether, various virulence and growth inhibitors have shown the potential to be developed as novel anti-APEC therapeutics. However, further efforts are needed to advance these inhibitors into field applications.

#### 4.6.3. Antimicrobial Peptides

Antimicrobial peptides (AMPs), regarded as new category of therapeutic agents, are short and generally positive charged peptides [226]. AMPs have fast and selective antimicrobial action, even against antibiotic-resistant bacteria, with low propensity for resistance development, which makes them ideal candidates for antibacterial development [226]. The protective effect of prophylactic in ovo treatment of D analog of chicken cathelicidin-2 (D-CATH-2; host defense peptide) was evaluated against APEC (O78:K80) infection in chickens [219]. The treatment of D-CATH-2 reduced the mortality (by 63%) and bacterial load (>90%) along with the increment of IgM level and peripheral blood lymphocytes and heterophils. In another study, the efficacy of orally administered surfactin–amoxicillin combination was evaluated against APEC (O78) infection in chickens [216]. Surfactin (lipopeptide) combination (0.01 mg/g) enhanced the efficacy (significant decrease in mortality, APEC load, and APEC lesions compared to amoxicillin treatment alone) of otherwise ineffective amoxicillin. The treatment also resulted in upregulation of expression of pro-inflammatory and anti-inflammatory cytokine genes (IL-1β, TNF-α, IL-10 and IL-13). In other studies, peptides (A3, P5, cecropin A-D-Asn, cLF36) have shown efficacy in decreasing the *E. coli* load in the chicken gut [30,227]. These studies demonstrate that antimicrobial peptides can be developed either as an alternative to antibiotics or as an adjuvant to antibiotics to enhance the efficacy of antibiotics.

## 5. Conclusions and Future Perspectives

APEC is a most common bacterial pathogen of poultry that causes significant economic losses to the poultry industry worldwide. The effective control of APEC is beneficial to both animal and human health. Multiple virulence and pathogenesis factors of APEC are involved in a coordinated way to cause systemic infections in poultry; therefore, a holistic approach encompassing all factors such as iron acquisition systems, QS system, bacterial metabolism, and secretion systems is necessary to formulate effective anti-APEC therapeutics in the future. Further investigation is necessary to provide concrete evidence for zoonotic transmission of APEC to humans. APEC isolates, particularly belonging to ST95 and ST131 or O1, O2, and O18 serogroups might serve as a source of human extra-intestinal infections. As a result of the significant antibiotic resistance issues and high risk of transmission of antibiotic-resistant bacteria and genes to humans, the development of antibacterials solely for animal uses without cross-resistance to current antibiotics might provide a solution for the future. Furthermore, there is a need for an ideal APEC vaccine that can provide cross-protection against multiple APEC serotypes. Knowledge gained on virulence and pathogenesis mechanisms of APEC should be exploited to identify the new vaccine candidates. Lastly, alternative therapies should also be considered for further development, especially the small molecule virulence inhibitors or growth inhibitors and AMPs with novel targets that could be effective at controlling colibacillosis in poultry.

## Figures and Tables

**Table 1:** APEC virulence and pathogenesis factors and their role in systemic infections.

Virulence Factors	Genes/Proteins Involved	Role in Pathogenesis/Infection	Reference
Adhesins	*fim*H, *fim*C, *pap*A, *pap*C, *pap*EF, *pap*G I, *pap*G II, *pap*GIII, *fel*A, sfa/*sfa*S, *afa*IBC, *foc*GE, *lpf*A, *lpf*0141, *lpf*0154, *flg*E, *crl*, *csg*, *bma*E, *tsh*, *mat*/*ecp*A, *hra*/*hrl*A/*hek*, *iha*, *yqi*G, *kii*	Adhesion, colonization, biofilm formation, motility, intracellular survival	[32,33,34,35,36,37,38,39,41]
	* yfc * O	Adhesion, colonization, resistance to environmental stresses	[42]
	* yad * C	Adhesion, intracellular survival, motility	[43]
	* aat * A, *aat*B, *upa*B	Adhesion, colonization, biofilm formation	[44,45]
	*fdt*A, *rlu*D, *yjh*B, *ecp*R, *fde*C	Adhesion	[46]
Invasins	*ibe*A, *ibe*B, *tia*, *gim*B	Invasion, resistance to oxidative stress, colonization, proliferation, biofilm formation	[35,47,48]
	IbeR	Invasion, resistance to serum and environmental stresses, expression of virulence genes	[49]
	* ych * O	Motility, adhesion, invasion, biofilm formation, expression of membrane proteins and metabolism genes	[50]
Iron acquisition systems	*iut*A, *iuc*C, *iuc*D, *aer*J, *iuc*A, *iuc*B, *iro*BCDEN, *fyu*A, *sit*ABCD, *mnt*H, *feo*B, *irp*2, *ire*A, *eit*ABCD, *fep*C, *chu*A, *bfr*	Iron and manganese uptake from the host, adhesion, invasion, colonization, persistence, expression of virulence genes, resistance to environmental stresses	[36,37,51,52,53,54,55,56,57]
	*ent*E, *ent*S, *tol*C	Invasion, colonization, persistence	[60]
Protectins	*iss*, *tra*T, *omp*T, *kps*MT(K1), *kps*MT(II), *kps*MT(III), *neu*C, *neu*S, *neu*D, *kfi*C-K5, *bet*A	Protect from serum bactericidal activity and phagocytosis, adhesion, invasion, intracellular survival, colonization, proliferation	[36,41,51,53,61]
	YbjX, PagP	Resistance to serum and environmental stresses, invasion, intracellular survival	[62,63]
	OmpA	Intracellular survival	[64]
	* wzy *	Adhesion, invasion, intracellular survival, colonization	[65]
	* waa * L	Motility, resistance to phagocytosis and environmental stresses, adhesion, invasion, biofilm formation	[65]
	* sod * A	Protect against ROS-mediated host defenses, biofilm formation	[68]
	* lpx * M	Invasion, intracellular survival, colonization, regulation of expression of cytokine genes and nitric oxide production	[67]
Toxins	*hly*F, *hly*A, *hly*E, *cdt*B, *cdt*S, *vat*, *sat*, *stx*2f, *ast*A, *pic*, EAST-1, *esp*C, *ace*4/35	Cell lysis and damage, induce host cell vacuolization, colonization, motility, biofilm formation, agglutination, formation of outer membrane vesicles	[35,38,39,41,51,53,69,70,71,72,73]
Other virulence and pathogenesis factors			
Quorum-sensing system (AI-2)	LuxS, LsrABCDFGK, *pts*I, Pfs	Motility, biofilm formation, adherence, invasion, colonization, intracellular survival, persistence, expression of virulence genes, cell damage	[10,74,75,76,77]
Secretion systems	DotU, CpxRA, IcmF, Hcp, ClpV, VrgG (Type VI)	Interbacterial competition, adhesion, invasion, intracellular survival, colonization, motility, biofilm formation, production of type 1 fimbriae, resistance to serum bactericidal activity, modulation of intracellular host responses (IL-18, IL-1β)	[11,80,81,82,83,84,85]
	EtrA, YqeI, EivC (Type III)	Motility, intracellular survival, resistance to phagocytosis and serum bactericidal activity, proliferation, expression of fimbriae genes, downregulation of pro-inflammatory cytokines	[12,78,79]
Two-component systems	PhoPQ, *tol*C	Biofilm formation, motility, adhesion, invasion, intracellular survival, systemic infection, expression of virulence genes and genes associated with flagellar assembly, ABC transporters, quorum sensing, and bacterial chemotaxis	[13,86,90]
	BasSR	Biofilm formation, APEC virulence and colonization *in vivo*	[91]
	KdpDE	Expression of flagella-related genes, flagellum formation, motility and resistance to serum bactericidal activity	[87]
	RstAB, *hde*D	Iron acquisition, acid resistance, intracellular survival, colonization	[88,92]
	BarA-UvrY	Adhesion, invasion, persistence, intracellular survival, resistance to serum bactericidal activity and oxidative stress, regulation of exopolysaccharide production and type 1 and P fimbriae	[89]
Transcriptional regulators	AutA/AutR	Expression of K1 capsule and acid resistance systems, adaptive lifestyle change	[14]
	FNR	Adhesion, invasion, expression of type 1 fimbriae and type VII secretion system, resistance to oxidative stress	[15]
	YjjQ	Flagellar motility	[93]
	McbR	Biofilm formation, response to H_2_O_2_	[94]
	*tyr*R	Invasion, motility, intracellular survival	[37]
	RfaH	Invasion, intracellular survival, resistance to serum bactericidal activity	[95]
Metabolism-associated genes	* acs * -*yjc*H-*act*P	Intracellular survival, proliferation, colonization, production of pro-inflammatory cytokines and nitric oxide	[96]
	PotE, PotF	Colonization, adhesion	[16]
	NirC	Adhesion, colonization	[17]
	ArcA	Chemotaxis, motility	[18]
Miscellaneous	OmpF, OmpC	Adhesion, invasion, colonization, proliferation	[100]
	Prophage phiv142-3 (*orf*20) and phiv205-1	Resistance to serum and environmental stresses, adhesion, invasion, intracellular survival, colonization, biofilm formation, formation of flagella and I fimbriae	[97,98,99]
	YicS	Motility, biofilm formation, invasion	[101]
	* cpd * B	Colonization	[102]
	* pst * B	Resistance to serum bactericidal activity and oxidative stress, colonization	[103]
	tmRNA-SmpB	Colonization, persistence, replication, intracellular survival	[104]
	* mli * C	Resistance to serum bactericidal activity	[105]
	*mal*X, *frz*, *cva*ABC, *cvi*, *cba*, *cib*/*cib*I, *cbi*, *cma*, *eae*A, *sop*B, *yfc*V, *gad*, *mch*BCF, *mcm*A, *bor*, *air*, eilA, *cel*B, *pab*B, *cap*U, *cif*, *tir*, *tccp*, *nle*B, *ia*L, *cjr*C, *mig-14p*	Unknown/not clearly known functions	[34,39,40,41,53,54,57,106,107,108]
Genes essential for systemic infections and adaptation	*met*H, *lys*A, *pnt*A, *pur*L, *ser*S, *ybj*E, *ycd*K (*rut*C), *wca*J, *gsp*L, *sds*R, *irp*2, *eit*D, *ylb*E, *yji*Y, *tkt*1, *pil*N, *pil*Q, *tsh*, *hpb*, TcfD, Z5222, *waa*O, *waa*Y, *iut*A, *iuc*A, *iuc*D, *iro*C, ColE2, *tra*K, *tra*G, *tra*T, *Sop*A, *psi*A, *hka*G, *hkb*V, *hkb*Q, Z3370, Int, CC0532, TM0427, YPO3000, *rhs*H, RSp0733, *bio*ABFCD, *rnf*A, *rfn*E, gene encoding endonuclease III, *cre*ABCD, *yeh*D, *pot*F, *flg*E, *tyr*R, *bfr*	Systemic APEC infections and adaptation	[37,109,110,111]

**Table 2:** Antibiotic resistance reported in APEC isolates worldwide from 2015 to 2020.

Study Location	Study Samples	Resistance Reported	Resistance Genes Reported	Reference
Belgium	Broilers	EFX	*gyr*A, *par*C, *par*E, PMQR	[172]
Nepal	Chickens	CLT, TTC, CFX, CXT, IPM	*mcr-1*	[171]
Greece	Layers	CLT	*mcr-1*	[170]
Bangladesh	Chickens	APC, DXC, TTC, NFN, CFX, NDA, CXT, IPM, GNC, CMC, SFN, AZT, PMB		[108]
Brazil	Chickens	CLT	*mcr*-5, *mcr*-9	[168]
Bangladesh	Layers	APC, TTC, CMC, EHC, EFX, CFX, SPM, CLT, GNC		[167]
Thailand and Australia	Broilers and broiler breeders	AXC, CFU, CMC, EFX, FFC, GNC, NDA, TMP-SFM, TTC	*gyr*A, *par*C	[51]
Tunisia	Chicken’s fresh carcasses	NDA, FMQ, EFX, DXC, TTC, FFC, TMP-SFM, SPM, AXC, AXC-CVA; CFT, CFZ, ATN, CFP, CLT	*mcr*-1, *bla*_CTX-M-1_, *bla*_TEM,_*bla*_SHV_, *tet*A, *dfra*I, *flo*R, *cml*A, *aad*A, *str*A, *str*B, *sul*1	[146]
Japan	Municipal wastewater Influents	CLT	mcr-1	[107]
Egypt	Broilers	APC, AXC-CVA, TTC, CLT, DXC, SMC, FFC, CFT, CFX		[32]
Korea	Broilers	APC, NDA, TTC, CPT, CPX	*bla*_CTX-M-1_, *bla*_CTX-M-15,_*bla*_TEM-1,_*aac*(3)-II, *qnr*A, *qnr*S	[147]
Germany	Broiler	CLT	*mcr*-1	[164]
Algeria	Broilers	NDA, AXC, APC, TCC, PPA, TMP-SFM		[148]
China	Chickens	APC, CFT, CMC, GNC, KMC, SPM, TMP-SFM, NDA, TTC		[53]
Taiwan	Day-old hatchery chicks	APC, AXC, CPX, FFC, TMP-SFM		[70]
Jordan	Broilers	TMP-SFM, FFC, AXC, DXC, SCC	*int*1, *sul*1, *sul*2, *tet*A, *bla*_TEM_	[71]
Italy	Broilers/turkey /goose/guinea fowl/duck/ pigeon/layers /capon	APC, NDA, AXC-CVA, CFT, CFX, CFN, CFZ, CMC, EFX, GNC, KMC, NDA, SPM, TTC, FMQ, TMP-SFM		[72]
France	Broilers/ducks /turkeys/layers	CFU		[165]
Ethiopia	Backyard chickens	APC, CFT, CXC, CRX, TTC		[149]
Pakistan	Broilers	APC, TTC, CFX		[34]
Indonesia	Chickens	TTC, OTC, CFX, CFX, NFX	*tet*A, *tet*B, *qnr*A, *qnr*B, *qnr*S	[150]
Canada	Broilers /Breeders	TTC, APC, SCC, GNC, CFU, KMC, APC, TMP-SFM		[54]
Egypt	Broilers	OTC, KMC, APC, CDC, SPM, EFX, CMC, CFT, GNC, EHC, OXC, TMP-SFM	CITM, *ere*, *aac*(3)-(IV), *tet*A, *tet*B, *dfr*(A1), *aad* (A1)	[151]
China	Layer	CRX, TMC, MMC, CFN, SPM, APC	*str*A, *str*B, *bla*_CMY-2_, *bla*_CTX-M-19_, *bla*_TEM-1B_, *fos*A, *mph*A, *flo*R, *sul*2, *tet*A, *tet*B	[152]
Senegal	Chickens	TTC, SSX, APC, TMP-SFM, SPM, CFU, CTX, CFX	*gyr*B, *par*C, *bla*_CTX-M,_*bla*_OXA-1_, *bla*_TEM,_*bla*_SHV_, *tet*A, *dhrf*VII	[153]
Algeria	Broilers	TTC, TMP-SFM, EFX, NDA, APC, DXC, CMC, AXC-CVA, FMQ		[166]
Brazil	Turkeys	EHC, APC, TTC, OTC, LCC, SCC, TMP-SFM	*bla*_CTX-M-2,_*bla*_CTX-M-8_, *bla*_CTX-M-8/25_, *bla*_TEM,_*bla*_SHV_, *bla*_CMY-2_	[154]
Nepal	Broilers	APC, CMX, DXC, CLT		[155]
Egypt	Broilers	PNC, CFP, NFX, CMC	*sul*1, *bla*_TEM,_*bla*_SHV,_*tet*A, *aad*A	[157]
Pakistan	Broilers	CLT	*mcr*-1	[156]
China	Chickens	TTC, NDA, TMP-SFM, APC, AKC, ATN, CFZ, CFT, CMC, CFX		[158]
Japan	Broilers	APC, CFT, OTC, SPM, NDA, EFX, TMP, KMC, GNC, CMC	*bla*_CTX-M-2,_*bla*_CTX-M-14_, *bla*_CTX-M-65_, *bla*_TEM-1,_*bla*_SHV-12_, *bla*_CMY-2_, *aac*A4, *aad*A2, *aad*A5, *cat*B3, *dfr*A1, *dfr*A17, *par*C, *aac*(6′)-Ib-cr, *oqx*AB	[159]
China/ Egypt	Chickens	CLT	*mcr*-1	[160]
Brazil	Chickens	APC, TTC, GNC, NMC, EFX, NFX, TMP-SFM		[35]
South Africa	Broilers	CLT	*mcr*-1	[161]
Spain	Broilers	CFT, CFZ, XCT, NDA, CFX, TTC, KMC, SPM, TMP-SFM, GNC, APC, FFC	*bla*_CTX-M-14_, *bla*_SHV-2_, *bla*_CMY-2_, *qnr*A, *aac*(6′)-Ib-cr	[162]
China	Chickens	TTC, NDA, APC, SFZ, SPM, TMP, CMX		[73]
Italy	Broilers/layers /turkeys	SPM, TMP	*aad*A *dfr*A	[163]
China	Broilers	APC, KMC, CFX, LFX, SPM, GNC, OFX, NFX, CTX	*bla*_CTX-M-15_, *bla*_OXA-30_, *bla*_TEM_, *bla*_SHV-2_, *bla*_CMY-2_, *qnr*A, *qnr*B, *qnr*S, *str*A, *str*B, *aph*(3′)-II1, *aac*(3)-IIa, *aac*(6′)-Ib, *ant*(3″)-Ia	[169]

NDA: nalidixic acid; FMQ: flumequine; EFX: enrofloxacin; CFX: ciprofloxacin; NFX: norfloxacin; LFX: levofloxacin; OFX: ofloxacin; DXC: doxycycline, TTC: tetracycline; OTC: oxytetracycline; FFC: florfenicol; TMP-SFM: trimethoprim-sulfamethoxazole; SSX: sulfisoxazole; SFZ: sulphafurazole; SFN: sulfonamide, CMX: co-trimoxazole; SPM: streptomycin; SMC: spiramycin; GNC: gentamicin; AKC: amikacin; KMC: kanamycin; SCC: spectinomycin; LCC: lincomycin; APC: apramycin; CDC: clindamycin; EHC: erythromycin; TMC: tobramycin; MMC: medemycin; NMC: neomycin; AZT: azithromycin; PNC: penicillin; APC: ampicillin; AXC: amoxicillin; TTC: ticarcillin; CXC: cloxacillin; OXC: oxacillin; AXC-CVA: amoxicillin-clavunilic acid; CFT: cefotaxime; CFZ: ceftazidime; CPT: cephatholin; CPX: cephalexin; CXT: cefoxitin; CFN: cefazolin; CRX: cefuroxime; CFU: ceftiofur; CTX: ceftriaxone; ATN: aztreonam; CFP: cefepime; CLT: colistin; PPA: pipedimic acid; CMC: chloramphenicol; NFN: nitrofurantoin; PMB: polymyxin B; IPM: imipenem.

**Table 3:** Various vaccine candidates tested against APEC infections in chickens.

Vaccine Description	Challenge	Route	Main Findings	Reference
Live-attenuated APEC O78 Δ*aro*A (Poulvac^®^ *E. coli*) and autogenous (O18, O78, and O111) vaccine (alone and combination)	Layers (O78, O18, and O111)	Spray, I/M	-Combination of live- attenuated and autogenous vaccine significantly reduce the mortality, lesion scores, and APEC load -Live-attenuated vaccine alone does not provide significant protection	[202]
Live-attenuated *Salmonella-*delivered vaccine secreting APEC antigens (PapA, PapG, IutA, ClpG)	Layers (JOL718)	Oral	-High IgA, IFN- γ, IL-2, and IL-6 levels -Complete protection against JOL718 challenge -Less lesions in liver	[182]
Live-attenuated APEC O78 Δ*aro*A vaccine (Poulvac^®^ *E. coli*) and formalin-inactivated APEC O78 bacterin	Turkeys (EC34195nal^®^, O78:K80)	Spray and S/C, respectively	-2 log10 CFU/g reduction in APEC load in spleen, liver, and kidney -No lesions -Induce transcription of IL-13 and TGF-β4 -Repress transcription of IFN- γ -Modulate CXCLi2 expression -Elevate IgA and IgY levels -Higher ability of splenocytes to proliferate	[183]
Live-attenuated APEC O78 Δ*aro*A vaccine (Poulvac^®^ *E. coli*)	Broilers	Spray	-High CD4^+^TCRVβ1^+^ cells -High naïve and memory CD8 cells -Stimulate high state of immunocompetence -Stimulate production of IgA	[190]
Live-attenuated Δ*ton*B and Δ*ton*B/Δ*fur* vaccine (E956)	Layers (E956)	Spray	-Less lesions In airsacs, heart, and liver	[184]
Siderophore receptor and porin (SRP^®^) APEC vaccine for egg layer peritonitis	Layers (O1, O2, and O78)	I/V, I/VAG, I/T, I/P	-Complete protection against mortality - Substantially reduce APEC colonization and lesions	[206]
Live-attenuated O78:K80 Δ*aroA* vaccine	Chickens and turkeys (O78 and X)	Spray or spray followed by oral booster	-Significant reduction in mortality -homologous and heterologous protection	[185]
Live-attenuated O78:K80 Δ*aroA* vaccine	Broilers (χ1378 and 02:K12)	Aerosol	-High weight gain -No antibody responses -No homologous and heterologous protection	[189]
Live-attenuated APEC O78:J29—Δ*crp* vaccine (AESN1331)	Layers (O78:J46)	Spray, eye drop, and in ovo	-Significant reduction in mortality -Less lesion scores in heart and liver	[186]
Live-attenuated *S*. typhimurium vaccine expressing O78 LPS and *E*. *coli* type 1 fimbriae	Layers (χ7122, χ7252, and χ7096)	Oral and spray	-Lower lesion scores in airsacs -No heterologous protection	[194]
Δ*gal*E, Δ*pur*A, and Δ*aro*A (APECO78:EC99) vaccine	Broilers (EC99 and EC317)	Spray	-High IgY level -Less lesion scores and APEC load -No heterologous protection	[196]
Live *E*. *coli* vaccine with *rsp*L mutation (EC844)	Broilers (EC317)	Aerosol and oral	-Lower APEC lesions with three doses	[200]
Recombinant *Salmonella* vaccine strain S740 (pSS28) containing APEC O1 and APEC O2 O-antigens	Layers (O1 C24-2, O2 CE37)	Oral followed by I/M booster	-High IgG and IgA levels -73.33% and 66.67% protection against O2 CE37 and O1 C24-2 challenge, respectively -High opsonophagocytosis and serum bactericidal activity -Less lesions in liver and spleen	[180]
Recombinant antigen (rAg) vaccine containing ExPEC proteins (OmpA, OmpT, TraT, EtsC)	Layers (O2)	S/C	-Higher IgY, IL-1β, IL-6, IL-18, IFN-γ, IL-4, IFN-β, and IL-8 levels -Increased serum bactericidal activity against multiple APEC strains -Less lesions in airsacs, liver, and heart -Less APEC load in heart and liver	[181]
Recombinant attenuated *S*. Typhimurium vaccine (RASV) χ9373	Layers χ7122 (O78:K80)	Oral	-High IgY level -Lower signs of airsacculitis -Lower lesion scores in heart and liver -Lower APEC load in blood -Increase blood and serum bactericidal activity against multiple APEC strains	[175]
Recombinant *Salmonella* vaccine strain S740 containing APEC O1 O-antigen (pSS27)	Layers (O1:C24-2)	Oral followed by and I/M booster	-High IgG and IgA levels ->50% protection against O1:C24-2 challenge	[191]
Recombinant *iss* vaccine	Broilers (O1, O2, and χ7122)	I/M	-High IgA and IgG levels -Less lesions in heart, liver, and airsacs -Heterologous protection	[188]
Recombinant *iss* vaccine	Layers (O2 and O78)	S/C	-High humoral response -Lower APEC lesions -Heterologous protection	[195]
*E*. *coli* BL21:D3 expressing FepA, FhuE, IroN, and IutA antigens	Broilers (CH2)	I/N	-High IgG level -No protection against CH2 challenge	[198]
*Lactobacillus saerimineri* expressing O78 FimC and OmpC antigens	Layers (O78)	Oral	-High IgG and IgA levels -Lower mortality	[199]
APEC O78, O1, and O2 outer membrane vesicles (OMVs)	Broilers (O78, O1, and O2)	I/M	-High IgG level -High IFN-γ, IL-17, and IL-10 levels ->90% protective efficacy -Reduce bacterial burden in liver and lung -No effect on growth performance	[201]
APEC O78 OMVs	Layers (O78)	I/M	-High IgG level -High opsonophagocytosis and serum bactericidal activity -Complete protection against O78 challenge	[176]
APEC O2 OMVs	Broilers (O2)	I/M	-Protective against homologous infection -Enhance specific (IgY) antibody response -Elicit IFN-γ mediated Th1 responses	[203]
Bacterial ghost vaccine (O2:K1) constructed using *Phi*X 174 lysis gene *E*	Broilers (O2:K1)	S/C, spray	-Less lesion scores in airsacs, liver, and heart -High IgG and sIgA levels -High IFN-γ	[177]
Bacterial ghost vaccine (O2: DE17Δ*lux*SΔ*aro*A) constructed using *Phi*X 174 lysis gene *E*	Layers (O2: DE17)	S/C	-Complete protection against O2:DE17 challenge -Less protection against O2: CE35 challenge -No lesions in liver, heart, spleen, and lung -Higher IgG, IFN-γ, and TNF-α levels	[178]
Bacterial ghost vaccine (O78:K80) constructed using *Phi*X 174 lysis gene *E*	Broilers (O78:K80)	S/C, spray	-Reduce lesions in airsacs, liver, and heart -High IgY, IgA, and IFN-γ levels	[179]
Bacterial ghost vaccine (O78:K80) containing E-lysis and S nuclease genes	Broilers (O78:K80)	S/C, spray	-Lower lesion scores -High antibody (IgY and IgA) and IFN-γ levels -Increase proinflammatory (IL-6, IL-1 β, and TNFSF15) cytokines production	[204]
Chitosan nanoparticles containing APEC O1 and O78 outer membrane proteins (OMPs) and flagellar antigens	Broilers (O1 and O78)	S/C	-High antibody titer ->80% protection against O1 and O78 challenges	[205]
PapGII196 vaccine	Broilers (CH2)	I/M	-High IgG level -No protection against CH2 challenge	[192]
FimH (FimH156) vaccine	Broilers (CH2)	I/M and I/N	-High IgG and IgA level -No protection against CH2 challenge	[193]
APEC O78:EC99 cell surface antigens (FimA, PapG, IutA, and LPS)	Broilers (EC99)	I/N	-High IgA, IgG, and IgM levels -Less mortality	[197]
Liposomal inactivated APEC O78:KAI-2 vaccine	Layers (APEC O78:PDI386)	Eye drop or spray	-High IgG and IgA levels -Less mortality, APEC load, and lesions	[187]

S/C: subcutaneous; I/M: intramuscular; I/P: intraperitoneal; I/N: intranasal; I/V: intravenous; I/VAG: intravaginal; I/T: intratracheal.

## Data Availability

Not applicable.
